# A real-world disproportionality analysis of apalutamide: data mining of the FDA adverse event reporting system

**DOI:** 10.3389/fphar.2023.1101861

**Published:** 2023-06-05

**Authors:** Zhihong Fang, Zhiqiang Xu, Wei Zhu, Mingming Yu, Chunmei Ji

**Affiliations:** ^1^ Department of General Surgery, The Affiliated Wuxi Children’s Hospital of Nanjing Medical University, Wuxi, Jiangsu, China; ^2^ Research Division of Clinical Pharmacology, The First Affiliated Hospital of Nanjing Medical University, Nanjing, Jiangsu, China; ^3^ Department of Urology, Shanghai Children’s Hospital, Shanghai Jiao Tong University, Shanghai, China

**Keywords:** apalutamide, FDA adverse event reporting system, disproportionality analyses, adverse event, real-world

## Abstract

**Background:** Apalutamide is a new drug class, which is approved to treat prostate cancer (PCa). The aim of our study was to assess the safety profiles of apalutamide in real-world through data mining of the United States Food and Drug Administration Adverse Event Reporting System (FAERS).

**Method:** We included adverse event (AE) reports regarding apalutamide submitted to the FAERS from 2018 quarter 1 (2018Q1) to 2022 quarter 1 (2022Q1). Disproportionality analyses, including reporting odds ratio (ROR), were performed to identify the signals of AEs in patients receiving apalutamide. A signal was detected if the lower limit of the 95% confidence interval (CI) of ROR >1 and at least 3 AEs were reported.

**Results:** The FAERS database documented 4,156 reports regarding apalutamide from 1 January 2018, to 31 March 2022. A total of 100 significant disproportionality preferred terms (PTs) were retained. Frequently observed AEs in patients receiving apalutamide included rash, fatigue, diarrhea, hot flush, fall, weight decreased, hypertension. The most significant system organ class (SOC) was “skin and subcutaneous tissue disorders”, which mainly consisted of dermatological adverse events (dAEs). The additional AEs observed with the significantly signal contain lichenoid keratosis, increased eosinophil count, bacterial pneumonia, pulmonary tuberculosis, hydronephrosis.

**Conclusion:** Our findings provide valuable evidence for apalutamide safety profile in the real-world, which could help clinicians and pharmacists to enhance their vigilance and improve the safety of apalutamide in clinical practice.

## 1 Introduction

Prostate cancer (PCa) is one of the most commonly diagnosed cancers in men and has the fifth highest mortality globally (Sung et al., 2021). Approximately 15%–30% of PCa patients may experience prostate-specific antigen (PSA) recurrence with radical treatment (Zumsteg et al., 2015; Cornford et al., 2021). Majority of these patients will receive androgen deprivation therapy (ADT) as initial treatment (Mohler and Antonarakis, 2019). However, long-term exposure to ADT eventually results in castration-resistant prostate cancer (CRPC), which contains metastatic (m) and nonmetastatic (nm) disease states (Karantanos et al., 2013; Mateo et al., 2019). The androgen receptor (AR) is overexpressed in such patients, which indicates that AR plays a central role in the pathogenesis of PCa. Studies have shown that direct inhibition of AR in addition to ADT may provide more complete blockade of androgen signaling than ADT alone (Clegg et al., 2012; Dai et al., 2017).

Apalutamide (Erleada^®^), an oral selective AR inhibitor, binds directly to the ligand-binding domain of AR. It impeded AR-mediated gene transcription and impaired nuclear localization and DNA binding in PC cells. Apalutamide has been approved for the treatment of nonmetastatic castration-resistant prostate cancer (nmCRPC) and metastatic castration-sensitive prostate cancer (mCSPC) in various countries (Al-Salama, 2019; Boukovala et al., 2020; Dror and Chi, 2020; Hoy, 2020). The recommended dose is 240 mg (four 60 mg tablets) administered orally once daily, and patients should also receive ADT. Meanwhile, the efficacy of apalutamide has been investigated recently. The large landmark randomized, double-blind, placebo-controlled clinical trials SPARTAN (NCT01946204) (Perez-Ruixo et al., 2020; Smith et al., 2021) and TITAN (NCT02489318) (Chi et al., 2019; Chi et al., 2021) confirmed that the addition of apalutamide to ADT prolongs metastasis-free survival and overall survival, maintained health-related quality of life, and the safety profile did not differ substantially from the placebo group. The most common AEs to apalutamide are fatigue, hypertension, rash, diarrhea, nausea, decreased weight, arthralgia, falls, hot flushes, decreased appetite, fracture, and peripheral edema.

Clinical trials are conducted under widely varying conditions, and AEs observed in the clinical trials of a drug may not reflect all AEs observed in practice. Thus, it is essential to evaluate the postmarketing safety profile of apalutamide in the real-world. The United States food and drug administration (FDA) adverse event reporting system (FAERS) is a well-known AE spontaneous reporting system (SRS), which can be employed to assess the potential association between drugs and AEs (Sakaeda et al., 2011; Michel et al., 2017). In this study, we aimed to explore the postmarketing safety profile of apalutamide based on AE reports from the FAERS database.

## 2 Materials and methods

### 2.1 Study design and data source

We extracted data from the FAERS database (http://www.fda.gov/) ranging from the first quarter of 2018 (2018Q1) to the first quarter of 2022 (2022Q1). The apalutamide was approved by the United States Food and Drug Administration (FDA) in February 2018, so the first quarter of 2018 was choosed as the start date. The generic and brand names (apalutamide, Erleada^®^) were used as keywords for data mining. Only reports documenting apalutamide as “primary suspect” or “secondary suspect” drug were included in our analysis. The AEs reports in FAERS database are coded with Preferred Terms (PTs) by the Medical Dictionary for Regulatory Activities (MedDRA). Tehierarchical structure of MedDRA allows grouping of PTs into relevant System Organ Class (SOC) which is the top level of MedDRA.

Only the reports which contain all three elements (identifiable patients, suspected drugs, and AE reports) were included in the present study. The duplicative reports were removed according to FDA guidelines, when case_id and fda_dt were the same, duplicate records under the same case were removed while keeping the latest fda_dt. Otherwise, AEs related to “product issues”, “medication and other product use errors”, “adverse event”, “death”, “social circumstances”, “prostatic neoplasms malignant”, “neoplasms benign”, “malignant benign”, “therapeutic procedures”, “product administration errors and issues”, “off-label use”, “drug ineffective”, and “disease progression” were excluded for which were not drug-related AEs.

### 2.2 Statistical analysis

Descriptive analysis was used to show the clinical characteristics of all AE reports associated with apalutamide from the FAERs database. Disproportionate analysis was performed to identify statistical associations between apalutamide and all AEs. The reporting odds ratio (ROR) were used to identify signals indicating a potentially increased risk of drug-associated AEs for apalutamide. A two-by-two contingency table was used to calculate ROR (Supplementary Table S1). A PT was considered positive singal if the lower limit of 95% CI was > 1, and the reported number was ≥ 3 (Sakaeda et al., 2013). The higher ROR inherently implies a stronger disproportion and strength signal, indicating that the specific drug is more likely to induce a specific AE than all other drugs (Zink et al., 2013).

## 3 Results

### 3.1 Population characteristics

The FAERs database received a total of 33461775 AE reports from 1 January 2018, to 31 March 2022, among which approximately 4,156 reports included 7,959 PTs for apalutamide. Patient characteristics and AE reports regarding apalutamide are presented in Table 1. Due to the specific indications, the patients were predominantly male (91.36%), while the sex of 8.64% of patients was unknown. Elderly patients (age > 65 years) contributed to the majority proportion of AE reports (47.96%), excluding unknown reports. Hospitalization-initial or prolonged (24.57%) was the most common serious outcome. AEs resulting in death were noted in 16.17% of reports, and the high proportion of deaths might be related to the disease progression of cancer. Most reports were submitted by physicians (23.56%) and consumers (32.36%). The United States (77.21%) represented the main source of reports, followed by Japan (10.08%). The number of reports increased yearly, except for the reports in the first quarter of 2022.

**TABLE 1:** Clinical characteristics of reports associated with apalutamide from the FAERs database.

Characteristics	Number(n)	Proportion (%)
Number of reports	4,156	
Sex		
Male	3,797	91.36
Unknown	359	8.64
Age(year)		
<45	1	0.02
45–64	176	4.23
65–74	640	15.40
≥ 75	1,353	32.56
Unknown	1,986	47.79
Serious outcomes		
Hospitalization-initial or prolonged (HO)	1,021	24.57
Disability (DS)	40	0.96
Life-threatening (LT)	102	2.45
Death (DE)	672	16.17
Other serious (OT)	1,100	26.47
Unknown	1,221	29.38
Reporting year		
2018	345	8.30
2019	891	21.44
2020	1,371	32.99
2021	1,049	25.24
2022Q1	500	12.03

### 3.2 Disproportionality analysis

The signal strength of AEs of apalutamide at the system organ class (SOC) level in the FAERS database is shown in Table 2. The frequently observed AEs in patients receiving apalutamide were referred to 20 organ systems. The most significant SOCs were “skin and subcutaneous tissue disorders”, “vascular disorders”, and “reproductive system and breast disorders”. Otherwise, the signals for “general disorders and administration site conditions”, “nervous system disorders” and “investigations” were also frequent and important.

**TABLE 2:** Signal strength of AEs of apalutamide at the system organ class (SOC) level in FAERS database.

SOC	N	ROR (95% CI)
Reproductive system and breast disorders	**28**	**3.12(2.15, 4.52)**
Vascular disorders	**296**	**2.87(2.55, 3.22)**
Skin and subcutaneous tissue disorders	**899**	**2.78(2.98, 3.19)**
Endocrine disorders	20	2.50 (1.61, 3.88)
Metabolism and nutrition disorders	197	2.26 (1.96, 2.61)
Investigations	518	1.81 (1.65, 1.98)
Cardiac disorders	192	1.68 (1.46, 1.94)
General disorders and administration site conditions	847	1.57 (1.47, 1.69)
Nervous system disorders	577	1.43 (1.31, 1.55)
Injury, poisoning and procedural complications	189	1.37 (1.18, 1.60)
Ear and labyrinth disorders	29	1.33 (0.92, 1.91)
Respiratory, thoracic and mediastinal disorders	213	1.17 (1.02, 1.34)
Blood and lymphatic system disorders	91	1.12 (0.91, 1.38)
Gastrointestinal disorders	502	1.08 (0.99, 1.18)
Musculoskeletal and connective tissue disorders	273	1.03 (0.91, 1.16)
Renal and urinary disorders	104	0.97 (0.80, 1.17)
Hepatobiliary disorders	27	0.97 (0.67, 1.42)
Infections and infestations	205	0.93 (0.81, 1.07)
Psychiatric disorders	188	0.84 (0.73, 0.98)
Eye disorders	24	0.76 (0.51, 1.13)
Immune system disorders	21	0.33 (0.21, 0.50)

The bold values are the most significant SOC.

Totally 100 PTs were detected as positive signals for apalutamide, which are presented in Table 3. Among thess signals, rash (PT:10037844), fatigue (PT:10016256), diarrhea (PT:10012735), hot flush (PT:10060800), fall (PT:10016173), weight decreased (PT:10047895), hypertension (PT:10020772) were the most common AEs, which were consistented with the manufacturer’s labeling and clinical trials. Some PTs with high ROR signals were found, including cerebrovascular accidents (ROR = 66.80, PT:10008190), exfoliative generalized dermatitis (ROR = 26.82, PT:10012456), increased blood thyroid stimulating hormone (ROR = 16.09, PT:10005833), increased blood testosterone (ROR = 23.55, PT:10005815), duodenal perforation (ROR = 25.28, PT:10013832), and right ventricular dysfunction (ROR = 18.05, PT:10058597). The additional observed AEs which were uncovered in the manufacturer’s labeling were found, such as lichenoid keratosis (ROR = 15.66, PT: 10064000), increased eosinophil count (ROR = 6.30, PT: 10064000), bacterial pneumonia (ROR = 6.03, PT: 10060946), pulmonary tuberculosis (ROR = 8.05, PT: 10037440), and hydronephrosis (ROR = 8.76, PT: 10020524).

**TABLE 3:** Signal strength of reports of apalutamide at the perferred terms (PTs) level in FAERs database.

	PT	N	ROR (95% CI)
**Expect AEs**	rash	444	8.25 (7.50, 9.07)
	fatigue	325	3.17 (2.84, 3.55)
	hot flush	**154**	**17.93(15.28, 21.04)**
	diarrhoea	151	1.79 (1.52, 2.10)
	fall	125	2.90 (2.43, 3.46)
	decreased appetite	122	4.04 (3.37, 4.83)
	weight decreased	108	2.77 (2.29, 3.35)
	dizziness	102	1.77 (1.46, 2.15)
	asthenia	95	2.06 (1.68, 2.52)
	oedema peripheral	93	2.14 (1.75, 2.63)
	arthralgia	87	1.64 (1.33, 2.03)
	hypertension	82	3.22 (2.59, 4.00)
	pruritus	73	1.67 (1.32, 2.10)
	acute coronary syndrome	54	3.44 (2.63, 4.50)
	interstitial lung disease	50	7.61 (5.76, 10.06)
	blood pressure increased	45	2.01 (1.50, 2.69)
	seizure	38	2.11 (1.53, 2.90)
	cerebrovascular accident	**35**	**66.80(47.80, 93.34)**
	balance disorder	32	2.82 (1.99, 3.99)
	hyperhidrosis	30	2.14 (1.49, 3.06)
	chest pain	30	2.35 (1.64, 3.37)
	dysgeusia	30	4.37 (3.05, 6.26)
	constipation	30	1.61 (1.12, 2.30)
	ischaemic stroke	29	2.96 (2.06, 4.27)
	cardiac failure	28	2.78 (1.92, 4.03)
	rash pruritic	27	4.56 (3.12, 6.65)
	dysphagia	27	2.51 (1.72, 3.66)
	rash erythematous	25	5.01 (3.38, 7.42)
	atrial fibrillation	25	2.07 (1.40, 3.07)
	toxic epidermal necrolysis	**24**	**14.46(9.68, 21.60)**
	blood thyroid stimulating hormone increased	**22**	**16.09(10.58, 24.47)**
	haematuria	22	3.12 (2.06, 4.75)
	dermatitis exfoliative generalised	**20**	**26.82(17.27,41.65)**
	drug eruption	**18**	**9.19(5.79, 14.60)**
	drug reaction with eosinophilia and systemic symptoms	17	4.55 (2.82, 7.32)
	taste disorder	16	6.04 (3.70, 9.87)
	urinary retention	16	4.05 (2.48, 6.62)
	rash maculo-papular	14	5.10 (3.02, 8.62)
	cardiac disorder	14	1.80 (1.07, 3.04)
	febrile neutropenia	14	1.70 (1.01, 2.87)
	cough	13	7.15 (4.15, 12.32)
	eczema	12	2.69 (1.53, 4.74)
	flatulence	12	1.80 (1.02, 3.18)
	hypokalaemia	12	2.07 (1.17, 3.64)
	hypothyroidism	12	3.04 (1.72, 5.35)
	Stevens-Johnson syndrome	11	5.66 (3.13, 10.24)
	hip fracture	11	2.86 (1.58, 5.16)
	dysuria	11	2.44 (1.35, 4.41)
	pollakisuria	11	2.18 (1.21, 3.94)
	ageusia	10	3.60 (1.93, 6.69)
	blood cholesterol increased	10	2.07 (1.11, 3.84)
	electrocardiogram qt prolonged	10	2.03 (1.09, 3.78)
	erythema multiforme	9	8.79 (4.57, 16.91)
	angina pectoris	7	2.17 (1.04, 4.56)
	gastric ulcer	6	2.42 (1.09, 5.40)
	hypertensive crisis	6	3.89 (1.75, 8.66)
	cardiac arrest	6	2.26 (1.01, 5.03)
	acute generalised exanthematous pustulosis	5	5.06 (2.10, 12.16)
	chest discomfort	**5**	**11.64(4.84,28.00)**
	generalised oedema	5	4.38 (1.82, 10.54)
	blood testosterone increased	**5**	**23.55(9.77, 56.73)**
	aortic dissection	**5**	**13.46(5.59, 32.40)**
	respiratory tract congestion	5	2.57 (1.07, 6.18)
	subdural haematoma	5	2.62 (1.09, 6.29)
	hyperthyroidism	5	2.48 (1.03, 5.95)
	subarachnoid haemorrhage	4	3.18 (1.19, 8.48)
	supraventricular tachycardia	4	3.97 (1.49, 10.58)
	ventricular extrasystoles	4	4.25 (1.59, 11.39)
	urine odour abnormal	4	5.13 (1.92, 13.67)
	dermatitis psoriasiform	**3**	**12.21(3.93, 37.93)**
	exfoliative rash	**3**	**11.54(3.72, 35.85)**
	pustular psoriasis	3	8.61 (2.77, 26.73)
	rash vesicular	3	5.12 (1.65, 15.89)
	duodenal perforation	**3**	**25.28(8.12, 78.67)**
	mucous stools	3	4.60 (1.48, 14.28)
	cytomegalovirus infection reactivation	3	8.33 (2.68, 25.87)
	heart valve incompetence	3	7.47 (2.41, 23.18)
	right ventricular dysfunction	**3**	**18.05(5.80, 56.10)**
	lumbar vertebral fracture	3	5.11 (1.65, 15.85)
	urinary bladder haemorrhage	**3**	**10.71(3.45, 33.26)**
	vanishing bile duct syndrome	**3**	**30.54(9.81, 95.11)**
**Unexpected AEs**	urinary tract infection	41	1.74 (1.28, 2.36)
	muscular weakness	23	1.68 (1.11, 2.53)
	Lethargy	16	2.68 (1.64, 4.38)
	Hallucination	15	1.69 (1.02, 2.81)
	Dementia	13	3.72 (2.16, 6.41)
	eosinophil count increased	8	6.30 (3.15, 12.62)
	Hydronephrosis	8	8.76 (4.38, 17.54)
	Agranulocytosis	8	3.83 (1.92, 7.67)
	pneumonia bacterial	7	6.03 (2.87, 12.67)
	abnormal dreams	7	3.00 (1.43, 6.30)
	restless legs syndrome	6	2.71 (1.22, 6.05)
	libido decreased	6	5.63 (2.53, 12.54)
	sensory disturbance	5	3.15 (1.31, 7.58)
	lichenoid keratosis	**4**	**15.66(5.87, 41.82)**
	muscle atrophy	4	3.01 (1.13, 8.02)
	pulmonary tuberculosis	4	8.05 (3.02, 21.48)
	Urosepsis	4	3.28 (1.23, 8.74)
	sjogren’s syndrome	3	4.14 (1.33, 12.85)
	middle ear effusion	3	9.89 (3.19, 30.72)

Expect AEs: predictable events on the basis of apalutamide mechanism of action or anticipated from pre-marketing pivotal trials with a safety signal. Unexpected AEs: unexpected or previously unreported events, not mentioned in the drug label. The bold values are the PTs with high ROR.

### 3.3 Time-to-onset analysis

**FIGURE 1:**
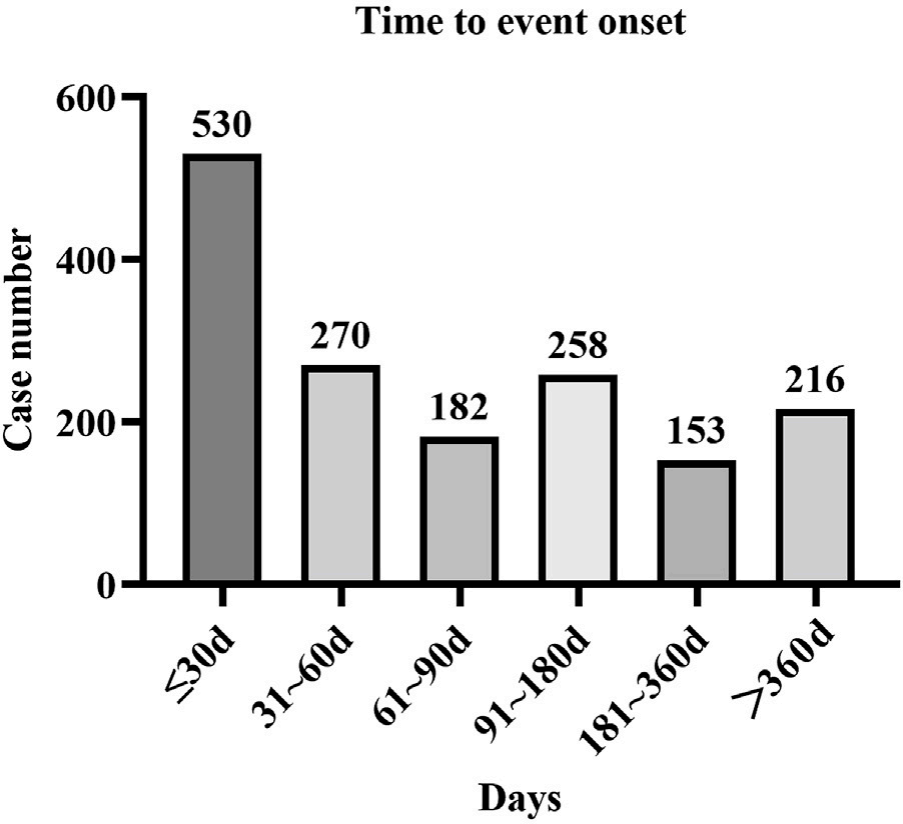
Time to onset of AEs in patients receiving apalutamide.

Approximately 1,609 AE reports were extracted from the FAERs database, which reported the onset time. The mean onset time was 126 days, and the median onset time was 62 days (interquartile range [IQR] 21–167 days). Our data demonstrated that the onset time of most AEs was less than 30 days (*n* = 530, 32.94%). Interestingly, AEs might still have occurred after 1 year for apalutamide treatment, with a proportion of 13.42% (Figure 1).

## 4 Discussion

Apalutamide, a high-affinity AR inhibitor, binds directly to the ligand-binding domain of AR and inhibits nuclear localization and DNA binding within prostate cancer cells (Al-Salama, 2018; Rathkopf and Scher, 2018). Apalutamide is a well-tolerated drug for PCa patients and is associated with a favorable trend of improved overall survival. The recent guidelines were updated to recommend apalutamide in patients with nmCRPC and mCSPC with background ADT therapy (Mohler and Antonarakis, 2019; Cornford et al., 2021). Thus, there may be an increasing annual trend of prescriptions, as well as growing clinical experience with apalutamide. It is important to underline monitoring safety and postmarketing surveillance for apalutamide. The present study provides real-world data on the safety profile of apalutamide.

We systematically reviewed the literature to evaluate the safety profiles of apalutamide. Ten clinical trial studies for apalutamide in PCa were identified (Supplementary Table S2) (Rathkopf et al., 2013; Smith et al., 2016; Rathkopf et al., 2017; Chi et al., 2019; Tsuchiya et al., 2019; Perez-Ruixo et al., 2020; Chi et al., 2021; McKay et al., 2021; Saad et al., 2021; Smith et al., 2021). The addition of apalutamide to ADT resulted in a safety profile that showed no substantial difference from placebo plus ADT. The most frequently investigated AEs were skin rash, fatigue, fracture, hypertension, hot flush, diarrhea, seizures, arthralgia and so on. Our disproportionality analyses revealed that the most common and frequently reported PTs for apalutamide were rash, fatigue, hot flush, diarrhea, falls, decreased appetite, decreased weight, and dizziness. The results were mostly consistent with the manufacturer’s labeling and clinical trials. We also identified that rash was more frequently reported, while falls, hypothyroidism, and seizures occurred in only a few patients.

Otherwise, our findings raise some different safety concerns. First, we detected some rare AEs with a high ROR, such as increased blood testosterone, duodenal perforation, and right ventricular dysfunction. Second, we found some unexpected PTs with high ROR, included lichenoid keratosis, increased eosinophil count, bacterial pneumonia, pulmonary tuberculosis, hydronephrosis. Notably, clinicians and pharmacists should be aware of these rare, new and additional observed AEs. Furthermore, no signals were detected following disproportionality for several frequently reported AEs listed on the drug label, such as nausea, vomiting, fracture, hematuria. These discrepancies could be explained by the fact that AEs are fairly common for all drugs in the FAERS database. Signal scores can be suppressed by a large number of reports for an AE associated with multiple drugs. Disproportionality requires that an AE is reported more (or less) frequently for a specific drug. The absence of a signal does not imply the absence of relative AEs; it only indicates that there was no disproportion seen for these AEs.

Our disproportionality analyses identified that the most common and significant SOCs for apalutamide were “skin and subcutaneous tissue disorders”. Interestingly, majority PTs belongs to “skin and subcutaneous tissue disorders” were dermatological adverse events (dAEs) (e.g., rash, pruritus, heperhidrosis). The SPARTAN and TITAN trials reported that the incidence of skin rash was 23.8% and 27.1%, respectively (Uemura et al., 2020). However, most skin rashes were grade 1–3 and rarely caused dose reduction or discontinuation. The highest frequency of dAEs commonly occurs 1–4 months post apalutamide initiation for PCa. Interestingly, the other AR inhibitor enzalutamide, darolutamine, showed a low rash incidence in clinical trials. This difference may be due to the chemical structure of apalutamide, which has a more reactive 2-cyanopyridine moiety and more readily activates the immune system by increasing lymph node cellularity and T cell and B cell counts (Drago et al., 2021; Pan et al., 2022). A published study showed that high apalutamide exposure was significantly associated with skin rash; thus, dose reductions may help prevent dAE recurrence (Perez-Ruixo et al., 2020). Most dAEs were effectively managed with moderate to high-potency topical steroids and oral antihistamines.

Pain and fatigue are common symptoms in metastatic PCa, while fatigue is also a common and substantial AE of ADT (Holm et al., 2018). Data mining from the FAERs database for apalutamide shows that fatigue is a frequently reported AE (ROR = 3.17). The *post hoc* analysis for the TITAN study showed that pain and fatigue were improved or not worsened in patients with mCSPC treated with apalutamide compared with placebo (Agarwal et al., 2021). The analysis demonstrated that patients benefitted through delayed disease progression, and they also maintained HRQoL with no additional pain or fatigue burden.

Due to the presence of incomplete reports in the FAER database, we could not identify the grade of the AE reports. Hence, the serious outcomes of AEs reports were explored in this study. About 44.15% AE reports suffered specific serious outcomes, including hospitalization-initial or prolonged (24.57%) and death (16.17%). The risk factors (such as weight, age, dose) for serious AEs was explored, however there was no sufficient clinical information to certify these. Hence, we remind clinicians and pharmacists that the prescriber’s information should be followed for managing AE related dose interruptions and/or modifications. Besides, we found that in the death reports most were related to the cardiovascular and cerebrovascular disease (a grouped term that included various events), excepted the reports related to the disease progression of cancer. Patients with clinically significant cardiovascular and cerebrovascular disease who are prescribed apalutamide should be monitored for risk factors (Morgans et al., 2021).

The results of this study indicated that the median onset time was 62 days, and most AEs occurred within the first month (*n* = 530, 32.94%) after exposure to apalutamide. The majority of AEs were reported within half a year, but AEs might still have occurred after 1 year. Thus, a longer follow-up period is needed to observe the AEs of apalutamide in future clinical studies.

Several limitations of the present study need to be addressed. The FAERs database is a spontaneous reporting database, so the rates of occurrence of each AE for apalutamide could not be estimated. Besides, the existence of a report does not establish causation in the FAERS database, so the results in the present study merely showed the potential AEs that meant the clinicians and pharmacists to enhance their vigilance. Meanwhile, the estimates of exposure adjusted AEs was not possible owing to incomplete information extracted from the FAERs database. The ROR was used as the measure of disproportionality, which provided the highest number of signals, which only report information on a possible causal relationship between an adverse event and a drug. Further well-organized clinical trials were need to investigate the causal relationship. Lastly, we could not distinguish AE reports related to apalutamide and concomitant ADT therapy owing to incomplete information. Hence, the association between concomitant ADT therapy and some related AEs could not be examined. Despite these limitations, the FAERs is still very useful for post-marketing safety surveillance.

## 5 Conclusion

In conclusion, the present study scientifically and systematically quantified the safety profile of apalutamide by the FAERS database. The frequent AEs (e.g.,, rash, fatigue, diarrhea, hot flush, fall) and additional observed AEs (e.g., lichenoid keratosis, increased eosinophil count, bacterial pneumonia, pulmonary tuberculosis) need to be monitored. Moreover, we observed strong signals for dermatological adverse events associated with apalutamide. Overall AE profile detected in this study is consistent with the clinical trial experience reported in the past and the manufacturer’s drug label for apalutamide. We hope that further studies and clinical practice will provide valuable evidence for the safety profile of apalutamide.

## Data Availability

The original contributions presented in the study are included in the article/Supplementary Material, further inquiries can be directed to the corresponding authors.
