# Supplementary material for: A real-world disproportionality analysis of apalutamide: data mining of the FDA adverse event reporting system
Source: Front Pharmacol. 2023 Jun 5;14:1101861. doi: 10.3389/fphar.2023.1101861 (PMC10277739; doi:10.3389/fphar.2023.1101861)
Supplement: Supplementary file 1 [file DataSheet1.doc]

**Supplementary material**

**Supplementary Table 1: Two-by-two contingency table.**

|  | AEs of interest | All other AEs | Total |
| --- | --- | --- | --- |
| Drug of interest | a | b | a +b |
| All other drugs in FAERs | c | d | c +d |
| Total | a+c | b+d | N=a+b+c+d |

**Notes:** a, number of reports containing both the suspect drug and the suspect adverse drug reaction; b, number of reports containing the suspect adverse drug reaction with other medications (except the drug of interest); c, number of reports containing the suspect drug with other adverse drug reactions (except the event of interest); d, number of reports containing other medications and other adverse drug reactions; N, number of all reports in the FAERS database.

ROR = (ad)/(bc); 95% CI = eln(ROR) ± 1.96(1/a + 1/b + 1/c + 1/d)^0.5; Criteria: 95% CI > 1, *N* ≥ 3

**Supplementary Table 2: Characteristics of the included apalutamide clinical studies studies.**

| **Author** | **Year** | **study name** | **ClinicalTrials.gov number** | **Study design** | **patients** | **Na** | **Np** | **Safty outcome** |
| --- | --- | --- | --- | --- | --- | --- | --- | --- |
| Chi KN et al | 2021  2019 | TITAN | NCT02489318 | Phase Ш | mCSPC | 526 | 526 | skin rush, fall, ischemic heart disease, ischemic cerebrovascular disorders, Hot flush, fatigue, hypertension,back pain, arthralgia, pain in an arm or leg, pruritus, weight increased, anamia, constipation, asthenia, bone pain, rash, generalized, blood alkaline phosphatase increased, urinary retention, fracture, hypothyroidism, seizure |
| Smith MR et al | 2020  2019 | SPARTAN | NCT01946204 | Phase Ш | nmCRPC | 806 | 401 | fatigue, hypertension, rash, diarrhea, nausea, weight loss, arthralgia, skin rush, fall, hypothyroidism, seizure,fracture, dizziness, |
| Rathkopf DE et al | 2013  2017 | / | NCT01171898 | Phase I | CRPC | 30 | / | fatigue, back pain, diarrhea, dyspnea, nausea, abdominal pain, arthralgia, constipation, headache, peripheral sensory neuropathy, musculoskeletal pain, pain in extremity, peripheral edema, hot flush, pain, hemorrhage |
| Smith MR | 2017 | / | NCT01171898 | Phase 2 | nmCRPC | 51 | / | fatigue, diarrhea, nausea, arthralgia, back pain, dysgeusia, hypothyroidism, hot flush, pain in extremity, cough, abdominal pain, decreased weight, pollakiuria, constipation, nasopharyngitis, hematuria, upper respiratory infection |
| Tsuchiya T et al | 2019 | / | NCT02162836 | Phase 1 | mCRPC | 6 | / | spinal-cord compression, renal disorder, abdominal discomfort, nasopharyngitis, dysgeusia, rash, hot flush |
| Saad fred et al | 2021 | ACIS | NCT02257736 | phase 3 | mCRPC | 492 | 490 | Hypertension, fall, skin rash, cardiac disorders, Hypokalaemia, Peripheral oedema, Fracture and osteoporosis, Ischaemic cerebrovascular disorders,Seizures |
| McKay RR et al | 2021 | / | NCT02903368 | Phase 2 | PC | 59 | 59 | hot flashes, fatigue, ALT increase, AST increase, hypertension, erectile dysfunction, insomina, maculopapular rash, headache, libido decreased, hypokalemia, urinary frequency, anemia, paychiatric disorder, diarrhea, anxiety |

**Abbreviation:** mCSPC, metastatic castration-sensitive prostate cancer; CRPC, castration-resistant prostate cancer; nmCRPC, nonmetastatic castration-resistant prostate cancer; mCRPC, metastatic castration-resistant prostate cancer.
